# Global lineage evolution pattern of sars-cov-2 in Africa, America, Europe, and Asia: A comparative analysis of variant clusters and their relevance across continents

**DOI:** 10.2478/jtim-2023-0118

**Published:** 2023-12-20

**Authors:** June Hyug Choi, Mee Sook Jun, Jeong Yong Jeon, Hae-Suk Kim, Yu Kyung Kim, Chang Ho Jeon, Seock Hwan Choi, Dong Sun Kim, Man-Hoon Han, Ji Won Oh

**Affiliations:** Department of Anatomy, BK21 FOUR KNU Convergence Educational Program of Biomedical Sciences for Creative Future Talents, School of Medicine, Kyungpook National University, Daegu, Republic of Korea; Department of Internal Medicine, Chungbuk National University, Cheongju, Yonsei University-Industry Foundation, Seoul, Republic of Korea; Absolute DNA, Inc. Daegu, Republic of Korea; Theragen Bio Co., Ltd., Seongnam-si, Republic of Korea; Department of Clinical Pathology, School of Medicine, Kyungpook National University, Daegu, Republic of Korea; Department of Laboratory Medicine, Catholic University of Daegu School of Medicine, Daegu, Republic of Korea; Department of Urology, School of Medicine, BioMedical Research Institute, Kyungpook National University Hospital, Kyungpook National University, Daegu, Republic of Korea; Department of Anatomy, BK21 FOUR KNU Convergence Educational Program of Biomedical Sciences for Creative Future Talents, School of Medicine, Kyungpook National University, Daegu, Republic of Korea; Department of Pathology, School of Medicine, BioMedical Research Institute, Kyungpook National University Hospital, Kyungpook National University, Daegu, Republic of Korea; Department of Anatomy, Brain Korea 21 PLUS Project for Medical Science, Yonsei University College of Medicine, Seoul, Republic of Korea

**Keywords:** lineage evolution, SARS-CoV-2, variant

## Abstract

**Objective:**

The objective of this study is to provide a comparative analysis of variant clusters and their relevance across Africa, America, Europe, and Asia, in order to understand the evolutionary patterns of the virus across different regions and to inform the development of targeted interventions and genomic surveillance eforts.

**Methods:**

The study analyzed the global lineage evolution pattern of 74, 075 severe acute respiratory syndrome coronavirus 2 (SARS-CoV-2) genomes from 32 countries across four continents, focusing on variant clusters and their relevance across regions. Variants were weighted according to their hierarchical level. The correlation between variants was visualized through Dimensionality reduction analysis and Pairwise Pearson's correlation. We presented a reconstructed phylogenetic tree based on correlation analysis and variant weights.

**Results:**

The analysis revealed that each continent had distinct variant clusters and different evolutionary patterns. The Americas had two clustered variants before lineage divergence and a downstream confluence lineage, Europe had bifurcation into two global lineages with an early occurrence of certain cluster while Asia had a downstream confluence of two large lineages diverging by two distinct clusters. Based on the cluster patterns of shared variants of the SARS-CoV-2 virus, Africa demonstrated a relatively clear distinction among three distinct regions.

**Conclusions:**

The study provides insights into the evolutionary patterns of SARS-CoV-2 and highlights the importance of international collaboration in tracking and responding to emerging variants. The study found that the global pandemic was driven by Omicron variants that evolved with significant differences between countries and regions, and with different patterns across continents.

## Introduction

The emergence and rapid spread of the severe acute respiratory syndrome coronavirus 2 (SARS-CoV-2) have caused a global pandemic with devastating consequences for public health and the global economy.^[1]^ SARS-CoV-2 is a positive-sense, single-stranded RNA virus that belongs to the family Coronaviridae.^[2]^ It is the causative agent of coronavirus disease 2019 (COVID-19), which has affected millions of people worldwide.^[3, 4]^ SARS-CoV-2 is thought to have originated in bats and was transmitted to humans through an intermediate host, possibly a pangolin. Since its initial identification in December 2019, SARS-CoV-2 has undergone rapid evolution, with several distinct lineages emerging worldwide. These lineages differ in their genetic makeup and have been associated with differences in disease severity, transmissibility, and vaccine efficacy.^[5]^ Understanding the lineage evolution of SARS-CoV-2 is critical for developing effective strategies to control the pandemic and prevent future outbreaks.

SARS-CoV-2 is an RNA virus that has undergone various evolutionary changes since its initial emergence in late 2019. Several genetic studies have found that the virus has a relatively slow mutation rate compared to other RNA viruses, which may be due to a proofreading mechanism that corrects errors during replication.^[6]^ Despite its slow mutation rate, SARS-CoV-2 has still undergone significant evolutionary changes that have contributed to its spread and ability to evade the immune system.^[7]^

Several factors have contributed to the emergence of new variants, including the high global prevalence of the virus, the large number of infected individuals, and the limited access to vaccines in many parts of the world.^[8]^ Additionally, the evolution of the virus is likely influenced by various host factors, such as individual immune responses and underlying health conditions.^[9]^ Efforts are underway to monitor and track the evolution of SARS-CoV-2, including through genomic surveillance programs that sequence the virus from infected individuals around the world.^[10]^ This information is used to track the emergence of new variants and inform public health strategies to control the spread of the virus.^[11]^

Tumors are known to evolve over time as a result of genetic mutations and natural selection, leading to the emergence of increasingly aggressive and treatment-resistant cancer cells.^[12]^ Similarly, viruses such as SARS-CoV-2 can undergo rapid evolution through mutations and natural selection, allowing them to adapt to changing host environments and potentially evade immune responses.^[13]^ By applying the principles of tumor evolution to the study of viral evolution, we can gain a deeper understanding of how SARS-CoV-2 evolves over time. This can have important implications for the development of vaccines and antiviral therapies, as well as our understanding of viral pathogenesis and the emergence of novel viral strains.

Phylogenetic inference can be employed to reconstruct clonal lineages and to comprehend tumor evolution by utilizing sequencing data.^[14, 15]^ In the case of phylogenetic trees, the common ancestors are represented by internal nodes, and their genotype can be inferred based on the similarities between their descendants.^[16]^ Hence, phylogenetic trees offer a way to investigate the past by determining the sequence in which mutations occurred as clones diverged in lineages and formed subpopulations.

The same principles used to study tumor evolution through phylogenetic inference can also be applied to the evolution of viruses, such as the SARS-CoV-2 virus. Viral evolution is driven by the same basic principles of natural selection and genetic drift as other organisms, with mutations accumulating over time and being passed down to subsequent generations.^[17]^ In the case of SARS-CoV-2, the virus has undergone multiple rounds of mutation and selection since its initial emergence in late 2019, resulting in the emergence of numerous viral variants with different characteristics.

Phylogenetic inference can be used to reconstruct the evolutionary history of the SARS-CoV-2 virus and track the emergence and spread of different viral variants. By analyzing the genetic sequences of different viral isolates and constructing a phylogenetic tree, researchers can estimate the order in which mutations occurred and identify the common ancestors of different viral lineages.^[18]^ This can provide important insights into the origins and transmission patterns of different viral variants, as well as their potential impact on disease severity and transmissibility.

One challenge in studying viral evolution is the rapid pace at which mutations can occur, as well as the potential for convergent evolution, where different viral lineages independently evolve similar mutations in response to similar selective pressures. However, the use of phylogenetic inference, combined with other methods such as experimental studies and epidemiological analysis, can help to provide a more complete picture of how the SARS-CoV-2 virus is evolving over time and how it may continue to evolve in the future.

In this paper, we explored the parallels between tumor evolution and SARS-CoV-2 evolution, highlighting the common mechanisms of genetic variation, natural selection, and adaptation. We applied concepts from tumor evolution to the study of viral evolution, including the use of phylogenetic analysis to track the spread of SARS-CoV-2 variants.

We analyzed the spread and evolution of 74, 075 SARS-CoV-2 sequencing data from multiple regions worldwide, including Africa, America, Europe, and Asia. The mutations accumulated over an extended period were analyzed sequentially through the tumor evolution method to reconstruct the temporal order of mutations. Moreover, we reconstructed groups of mutations that propagate through regions with locally similar mutations. This analysis shows that the virus evolution process shares similarities with the lineage evolution process observed in tumors. Our findings suggest that studying SARS-CoV-2 through the lens of tumor evolution can provide a better understanding of its evolutionary dynamics.

## Material and method

### Research plan

For this study, a comprehensive dataset of 74, 075 SARS-CoV-2 genomes from 32 countries across four continents, namely Africa, America, Europe, and Asia was involved analyzing. The dataset was analyzed using a combination of phylogenetic analysis and evaluation scores to integrate country-specific trees and reconstruct a worldwide lineage tree. The evaluation scores were based on the hierarchy of variant occurrence, with higher scores indicating more frequent and widespread variants. The evolutionary patterns of SARS-CoV-2 were analyzed using identification of distinct variant clusters and their relevance across different regions. Concepts from tumor evolution were applied to the study of viral evolution, including the use of phylogenetic analysis to track the spread of SARS-CoV-2 variants.

### Sequencing data of the SARS-CoV-2 and data analyses with visualization

To investigate the global pattern of SARS-CoV-2 mutations, phylogenetic lineage trees and total 74, 075 raw sequencing data were obtained from the GISAID database.^[20]^ The reported GRA nonsynonymous variants were identified and used to assemble the dataset. All sequencing data was provided as a consolidated source of hCoV-19 phylogenetic information, and ethical issues were not an issue as the patient source was not specified.

We employed various libraries for analysis and visualization. Data visualization was carried out using the seaborn library in Python, while data analysis employed the prcomp, Rtsne, and umap libraries in R. Dimensionality reduction techniques, specifically prcomp, Rtsne, and umap, were applied to project the complex relationships among the variants into two dimensions for visualization. This reduction facilitated a comprehensive visual examination of the variant relationships.

Weight assignment of variants based on the hierarchical positions of lineage trees.

To evaluate the importance of each variant in the phylogenetic lineage trees, exponential weights were assigned based on their hierarchical positions. The weight score was determined using the formula,


$$\text{ Weight score }=2^{c-\text{ event branch number }}$$

where c is a constant value for integer-derived weights and event branch number represents the branch number in the phylogenetic tree where the variant occurred. The weights were assigned separately for each country or region’s phylogenetic lineage tree. The exponential function allowed for the assignment of higher weights to variants that occurred at deeper hierarchical levels, indicating their greater importance in the evolutionary history of the virus.

The weight score assigned to each variant was used in subsequent analyses, such as the integration of phylogenetic trees and the reconstruction of a worldwide lineage tree.

### Dimensionality reduction analysis

In this study, three different dimensionality reduction approaches were utilized, namely Principal Component Analysis (PCA), t-Distributed Stochastic Neighbor Embedding (t-SNE), and Uniform Manifold Approximation and Projection (UMAP). We employed these approaches to reduce the dimensionality of the high-dimensional data by identifying the principal components that explained the maximum variance in the dataset. Each plot was visualized using prcomp, Rtsne, and umap libraries in R. These libraries were used to generate plots that show the distribution of variants in the lower-dimensional space, with each point representing a variant. The resulting plots provide a visual representation of the relationships between the variants and allow for the identification of clusters of variants that are closely related. Scripts and data tables for dimensionality reduction analysis are provided at GitHub (https://github.com/G1slab/COVID).

### Pairwise Pearson’s correlation

In this study, we used the approach to assess the relationships among different variants of the SARS-CoV-2 virus. To measure the distance between variables in the multidimensional space, we employed the Euclidean distance metric. This metric provided a means to quantify the proximity of variables in the multidimensional space, enabling us to compare the similarities and differences between different variants of the virus. We implemented the pairwise Pearson’s correlation approach in conjunction with the Euclidean distance metric to assess the linear relationships between variables of interest. The Euclidean distance (2-norm) served as the distance metric between the points.

To analyze the correlations between individual variants, we utilized the weight scores of the variants. Weight scores were assigned to the nonsynonymous variants in the phylogenetic lineage trees reported for each country or region based on their hierarchical positions. Exponential weights were assigned to these variants based on the hierarchical levels at which they occurred. To create the pairwise Pearson’s correlation plot, we used the seaborn library in Python. The plot allowed us to visualize the correlations between different variants of the virus.

### Evaluation and reconstruction of the order of variant emergence and phylogenetic lineage tree

To evaluate the order of variant emergence, we first identified the nonsynonymous variants in the phylogenetic lineage trees reported for each country or region. Next, we assigned exponential weights to the variants based on their hierarchical positions within the lineage trees, as described previously. We then computed the sum of weights for each variant that occurred within the lineage tree, providing a measure of its prevalence within the population.

To reconstruct the phylogenetic lineage tree, we considered the correlated variant group, determined by pairwise Pearson’s correlation analysis. This approach allowed us to accurately represent the relationships between the variants and to construct the lineage tree based on the order of variant emergence.


$$\text{ Estimation score }=\sum_{i=1}\text{ Weight }{\mathrm{score}}_{i}$$

The evaluation and reconstruction of the order of variant emergence and phylogenetic lineage tree were carried out using Rtsne and umap libraries in R. The R scripts used for the evaluation and reconstruction are available at the following GitHub repository (https://github.com/G1slab/COVID).

## Result

### Global evolutionary landscape of the SARS-CoV-2 virus

**Figure 1:**
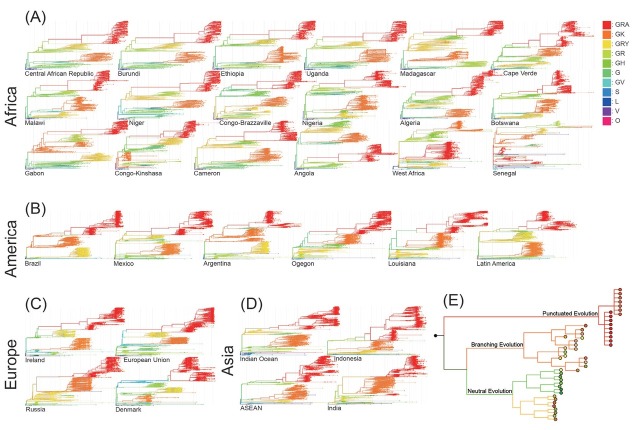
SARS-CoV-2 evolutionary lineages: (A) 18 nations or areas in Africa; (B) 6 nations or areas in the Americas; (C) 4 nations or areas in Europe; and (D) 4 nations or areas in Asia. Data regarding these lineages was sourced from GISAID. Classification of SARS-CoV-2 clades, referred GRA to O, is presented in Table 1. (E) Schematic lineage showed the three distinct patterns of virus evolution that arise throughout the progression of SARS-CoV-2.

From GISAID, we assembled and analyzed 32 SARS-CoV-2 virus phylogenetic lineage trees from 18 African, 6 American, 4 European, and 4 Asian regions (Figure 1).

Each tree ultimately culminated in omicron becoming the dominant species, and the patterns of evolution of the lineages were largely similar across regions in global view. In particular, the patterns of evolution of the lineages were similar to traditional tumor evolutionary lineage trees (Figure 1A-D). However, when examined over time, while tumor lineages survive by maintaining heterogeneity in the long term within tumor, in the case of the SARS-CoV-2 virus, each lineage disappeared in the final stages of the virus lineage, with new species emerging from other lineages.

Given the isolation of each region, it seems likely that the process of mutation between regions and even continents and the global pattern of lineage evolution in each region and the timing of divergence are similar. Nevertheless, we were able to identify the possibility of regionally independent variation. In particular, we found regions with atypical evolutionary trees, such as Senegal, West Africa, and Denmark (Figure 1A and C). They show an atypical pattern when analyzed with the available samples of the SARS-CoV-2 virus. The other regions are generally similar, which is noteworthy given the potential for a pandemic at the global level.

When analyzing their global patterns based on the clade classification of SARS-CoV-2 virus presented in Table 1, evolutionary patterns of the SARS-CoV-2 virus emerge from the perspective of cancer evolution, we found similar trends have been observed in the SARS-CoV-2 virus evolution.^[16, 19]^ For example, G, GH, GR, GRY proceeded with neutral evolution while GK generally showed a typical branching evolution. In particular, GRA (omicron), which appears to be typical of punctuated evolution. Punctuated tumor evolution is a pattern of clonal expansion in which early mutations survive their clonal sweep by slowly accumulating mutations. In the case of omicrons, we can see that many sub-lineage SARS-CoV-2 viruses accumulate mutations that are initially undetectable, slowly accumulating variation, and then suddenly exploding in population to become the dominant species in pandemic. (Figure 1E). It can be inferred that they have relatively many shared mutations and have survived clonal sweeping for a long period of time to reach their final state.

**Table 1:** Clade classification of SARS-Cov-2^[20]^

Clade	Context of marker variants
	C241T, C3037T, A23403G, G28882A includes S: D614G+N: G204R+(at least 6 of the following amino acid
GRA (Omicron)	changes S: V143del, Y145del, N211del, ins214EPE, G339D, S371L, S477N, T478K, E484A, Q493R, Q498R, T547K, N679K, P681H, P681R, N764K, D796Y, N856K, Q954H, N969K, L981F)
GRY	C241T, C3037T, 21765: 21770del, 21991: 21993del, A23063T, A23403G, G28882A includes S: H69del, V70del, Y144del, N501Y+D614G+N: G204R
GH	C241T, C3037T, A23403G, G25563T includes S: D614G+NS3: Q57H
GR	C241T, C3037T, A23403G, G28882A includes S: D614G+N: G204R
G	C241T, C3037T, A23403G includes S: D614G
GK	C241T, C3037T, A23403G, C22995A includes S: D614G+T478K
GV	C241T, C3037T, A23403G, C22227T includes S: D614G+A222V
S	C8782T, T28144C includes NS8: L84S
L	C241, C3037, A23403, C8782, G11083, G26144, T28144(early clade markers in WIV04: reference sequence)
V	G11083T, G26144T NSP6: L37F+NS3: G251V
O*	-

*Others.

### Global lineage evolution pattern of Africa

We conducted an analysis of the history of SARS-CoV-2 variants that occurred in different countries and regions, focusing on the evolution of the omicron variant. The punctuated evolution pattern of omicron, characterized by the accumulation of mutations over a relatively long period of time, suggests that clonal sweeping may have occurred. In tumor evolution, the ancestral lineage of shared mutations is often obscured by clonal sweeping, but we sought to identify evolutionary patterns in SARS-CoV-2 by comparing mutations across regions.

**Figure 2:**
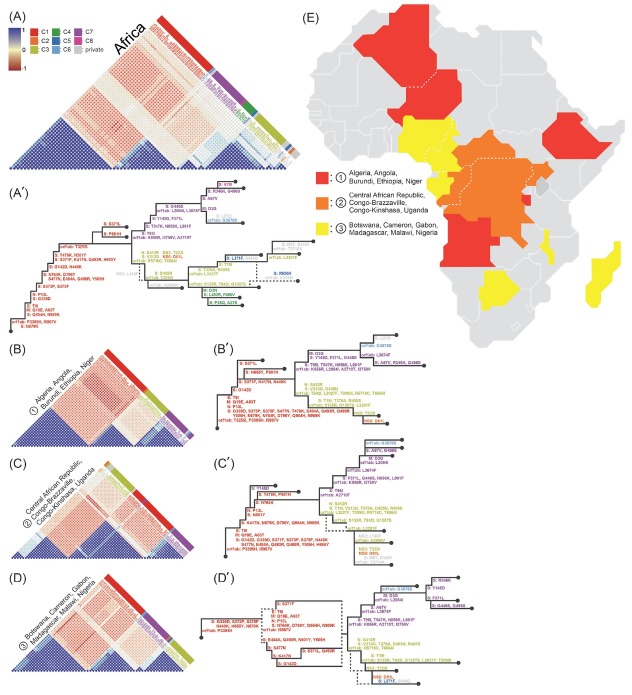
Clustering of 77 GRA variants and their pairwise Pearson’s correlation following hierarchical weight; (A) The correlation of SARS-CoV-2 variants in 18 distinct countries or regions of Africa. (B-D) Based on the relevance of SARS-CoV-2 lineages among African countries, the countries or regions were categorized into three distinct groups (Figure 4C). Pearson’s correlation analysis was performed using seaborn in Python and applied to hCoV-19 phylogenetic data available on GISAID. (A'-D') Reconstructed phylogenetic lineage trees based on variant correlations and hierarchical evaluation scores in Africa (Figure S1A). Dashed branches indicate lineages for which bifurcation discrimination is not possible. Correlated variants, manually sorted according to Pearson’s correlation based on worldwide variants information, exhibited uniform color-coding throughout all plots. Variants reported only once were categorized as private. (E) The countries classified into three groups were geographically mapped.

By integrating data from 18 African countries, we were able to infer the order of shared mutations from the earliest to the latest branching mutations, as shown in Figure 2A and A’. Through an integrated analysis of the degree of mutation sharing with other countries and globally, we identified eight clusters based on their interconnectivity. (Table 2) In the case of the red C1 cluster, the earliest mutations were observed in all African countries, and as the evolutionary march progressed, there were mutations exclusively found in each country.

**Table 2:** Clusters according to Pairwise Pearson’s correlation

Cluster	Mutation list
C1	S371L, N501Y, Q493R, S477N, Q498R, E484A, Y505H, G339D, N440K, S375F, S373P, T9I, A63T, Q954H, N969K, D796Y, N764K, I5967V, P3395H, P13L, H655Y, N679K, Q19E, K417N, S371F, P681H, G142D, T3255I, T478K
C2	D61L
C3	L3201F, T223I, T376A, D405N, R408S, L3027F, S413R, R5716C, T842I, S135R, G1307S, T19I, V213G, T6564I, T3090I
C4	F486V, D3N, L452R, P26Q, A27S
C5	L371F, F3677L, R856K
C6	G3676S, N211I
C7	Y145D, G446S, L2084I, F371L, K856R, D3G, R346K, G496S, A67V, L3674F, A2710T, T95I, I3758V, T547K, N856K, L981F
C8	Y144*, V70I
private*	R204G, S391F, S3675F, T2710A, I95T, D145Y, A2909V, P25T, S446G, V3758I, G3D, L212I, L106F, D343G, V143H, S2083N, L212V, T3646A, L2084V, S2083T, K440N, H69V, Y144L, G204R, S33G, R493Q

*Mutation that occurred only once in all phylogenetic trees.

Our analysis revealed three distinct patterns of variant occurrence within the African continent, which we designated as group 1 (Algeria, Angola, Burundi, Ethiopia, Niger), group 2 (Central African Republic, Congo-Brazzaville, Congo-Kinshasa, Uganda), and group 3 (Botswana, Cameron, Gabon, Madagascar, Malawi, Nigeria), as shown in Figure 2B-D. These groups were geographically distributed from north to south (Figure 2E), and shared the same initial variant occurrence, characterized by mutations in E: T9I, M: Q19E, A63T, S: G339D, S373P, S375F, and orf1ab: P3395H (Figure 2B’-D’). However, groups 1 and 3 were trend-separated, with group 2 bridging the trend in the middle (Figure 4C).

**Figure 3:**
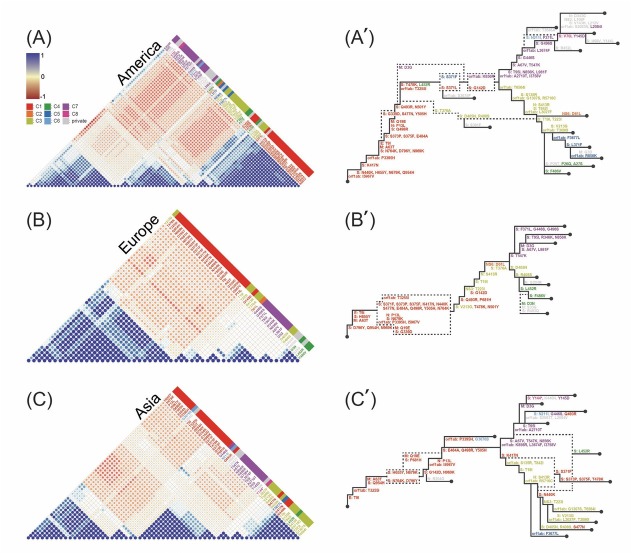
(A-C) Clustering of 92 GRA variants and their pairwise Pearson’s correlation following hierarchical weight. (A'-C') Reconstructed phylogenetic lineage trees based on variant correlations and hierarchical evaluation scores in each continent (Figure S1A). Dashed branches indicate lineages for which bifurcation discrimination is not possible. Correlated variants, manually sorted according to Pearson’s correlation based on worldwide variants information, exhibited uniform color-coding throughout all plots. Variants reported only once were categorized as private.

**Figure 4:**
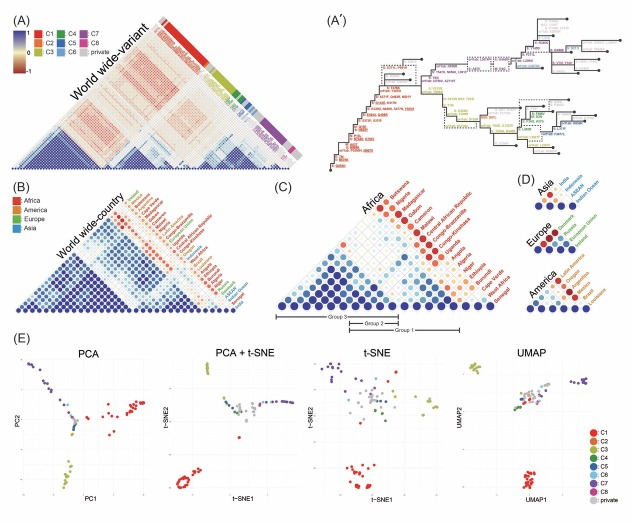
(A) Clustering of 99 GRA variants and their pairwise Pearson’s correlation following hierarchical weight and (A') reconstructed phylogenetic lineage tree on the basis of variant correlations and hierarchical evaluation scores in Worldwide (Figure S1B). Variants clustered on Pearson’s correlation were each indicated by different color. Variants that reported only a single time were labeled as private. The underline indicates a pre-branching mutation commonly founded across all continents. Clustering countries or regions according to relevance of hierarchical weight of GRA phylogenetic lineages; (B) 32 Worldwide; 18 Africa; (D) 4 Asia, 4 Europe and 6 America. According to the pairwise Pearson’s correlation, the three distinct groups were classified as Group 1-3. (E) Dimensionality reduction analysis visualized. The dataset used for executing all three dimensionality reduction approaches was derived from the SARS-CoV-2 phylogenetic information accessible on GISAID. The input data incorporated 99 GRA variants along with 32 phylogenies information originating from distinct countries or regions (Figure S1B). Variants, manually sorted according to Pearson’s correlation coefficient, exhibited uniform color-coding throughout all four plots.

The analysis suggests that even within the relatively large African continent, there is evidence of shared patterns of variant occurrence, possibly influenced by geographic proximity. Furthermore, the identification of shared mutations across regions may provide insight into the ancestry of SARS-CoV-2 variants, despite the occurrence of clonal sweeping.

### Global lineage evolution pattern of America, Europe, and Asia

We examined the pattern of Omicron evolutionary marching in three different continents. Characteristically, compared to Africa, each of the variant clusters in the three continents appeared significantly different (Figure 3A-C).

In the clustering based on each shared and privately observed mutation in the analyzed virus sequences, and in the reconstructed tree based on the evaluation scores, the Americas had clustered variants in C3 (yellow) and C7 (purple) that occurred before the lineage divergence, as well as a lineage that immediately joined the descendant lineage *via* the S: T375A, D405N, and R408S variants (Figure 3A’).

In Europe, the occurrence of the C1 (red) variant tended to bifurcate into two global lineages, with the C3 variant occurring relatively early, followed by the S: D405N and S: T547K variants (Figure 3B’), in contrast to lineage bifurcation with C3 and C7 in other continents.

In Asia, a large proportion of C1 variants were found downstream of the lineage divergence by C3 and C7. In addition, a lineage interpreted as a downstream confluence of two large lineages diverging by C3 and C7 occurred that was not present in reconstructed trees from other continents. In particular, the variants that determined the downstream lineage divergence were the C1 (K417N) and C8 variant clusters, unlike in other continents (Figure 3C’).

When analyzing the relevance of each country or region, there was a tendency for separation in terms of correlation, with two groups emerging in the Americas- Mexico, Argentina, Latin America, Oregon and Brazil, Louisiana; two groups in Europe- Denmark and Russia, European Union, Ireland; and two groups in Asia- Indonesia and ASEAN, Indian Ocean, India (Figure 4B and D). In summary, even within the same continent, countries like Denmark and Indonesia have relatively different patterns than other countries within the same continent. However, in comparison to Africa, the extent of clustering observed in these continents was relatively lower (Figure 4C). These analyses suggest that the omicron variants that drove the global pandemic (Figure 1) actually evolved with significant differences between countries and regions, and with different patterns across continents while they show the similar global branching patterns.

### Integrated global lineage evolution pattern of the SARS-CoV-2 virus

The integrated analysis of global mutation patterns was made possible by the results obtained from each continent. In the process of integration, we assigned an evaluation score from early to late depending on which hierarchy each variant occurred in the previously reported country-specific trees, which allowed us to integrate a total of 32 trees and divide them into eight correlated variant clusters (Figure 4A). The clusters derived from pairwise Pearson’s correlation centered on each country showed almost the same tendency under dimensionality reduction approaches (Figure 4E). A worldwide lineage tree was reconstructed by combining the evaluation scores and the relatedness between variants (Figure 4A’).

In the worldwide reconstructed tree, once all the variants in Cluster 1 occurred, it was estimated to diverge into two lineages based on the occurrence of the variants (S: T95I, orf1ab: I3758, A2710T) and (S: V213G, orf1ab: T6564I). While the sequence of variant occurrences in the reconstructed global tree was often perfectly ancestral, we also observed variants that appeared to form independent pathways, such as S: S371L, P681H, orf1ab: R5716C, and then rejoined at a later stage, or lineages that diverged, such as S: A67V, orf1ab: L3674F, and then rejoined at a later stage (Figure 4A’).

Intriguingly, when the worldwide lineage tree was reconstructed, the intra-cluster order of each continent was different (Figure 2A’). For example, globally, the S: Q954H variant was estimated to be the first to arise, while in Africa, three variants, S: N679K, orf1ab: P3395H, and I5967V, were estimated to be the first to arise (Figure 2A’ and Figure 4A’). In contrast, the detailed order of occurrence in each lineage was different, but the first variants to appear in the first lineage branch of all analyzed were the same:(S: T95I, orf1ab: I3758, A2710T) and (S: V213G, orf1ab: T6564I).

Furthermore, globally, Asia has a relatively isolated evolutionary lineage, while the other three continents share a lot in common, with countries such as Cape Verde, West Africa, Senegal, Denmark, and Indonesia having more or less independent evolutionary pathways regardless of their geographic proximity (Figure 2, Figure 3 and Figure 4B). Despite the differences in evolutionary patterns of GRA observed between continents or countries, it is noteworthy that 15 common mutations were observed across all continents prior to the lineage tree branching into C3 and C7 (Figure 4A’). These findings suggest the possibility of SARS-CoV-2 converging around vulnerable mutation sites, despite undergoing relatively independent evolutionary processes.

## Discussion

We conducted a comprehensive analysis of the SARS-CoV-2 virus phylogenetic lineage trees derived from 18 African, 6 American, 4 European, and 4 Asian regions using data from GISAID. Our analysis revealed that the patterns of evolution of the lineages leading to the emergence of omicron, the currently dominant species, were largely similar across regions in global view. However, it is clear that the virus evolved differently across different continents, with each region exhibiting its unique variant clusters and patterns while maintaining the global interaction leading to similar marching evolution. The integration of the results from each continent allowed us to identify eight correlated variant clusters, and the worldwide lineage tree was reconstructed based on the occurrence of variants and their relatedness.

The method of assigning weights based on the hierarchical positions of variants allowed for the evaluation of the global importance of each variant and its role in the evolution of SARS-CoV-2. It also enabled the identification of variants that were highly conserved across multiple regions or countries, as well as those that were unique to specific regions. The use of exponential weights also provided a more nuanced analysis of the contribution of each variant to the evolution of the virus, compared to a simple binary classification of the presence or absence of a variant in a given phylogenetic lineage tree.

These patterns of evolution were observed to be similar to those observed in traditional tumor evolutionary lineage trees,^[16, 21, 22]^ as shown in Figure 1. However, the SARSCoV-2 strains appeared to have diverged from the viral lineage in the final stages, unlike tumor lineages that maintain heterogeneity in the long term for survival. There are many reasons why a particular SARS-CoV-2 virus lineage may disappear, including the development of a vaccine or a treatment that works better for certain lineages or a lethal event that prevents further progression of infection.^[7, 23, 24]^ Unlike tumor clonal sweeping,^[25, 26, 27]^ in the case of the SARS-CoV-2 virus, a particular lineage may explode in infection at a particular time point and then suddenly disappear.^[28]^ It is not clear whether this is the case for tumors, but in cases with strong germline drivers in early neonatal cancer,^[29, 30]^ there is chance for each cancer lineage to become many different evolutionary forms of cancer in neutral or branching patterns.^[31]^ We did not observe the SARS-CoV-2 virus where a single lineage reemerged as a new pattern of lineages in the long term. This suggests that further research is needed to determine if there are any patterns of SARS-CoV-2 virus that can survive in the long term without causing the severe pathological event rather, it is a form of infectivity that survives in an individual or inter-individual for a long period of time and remains infectious.

Our analysis is essentially based on the similarity between the characteristics of a tumor that has undergone many different sweeps over time and the characteristics of a long-term latent SARS-CoV-2 virus. We hypothesized that this could potentially allow for attempts to explain the evolutionary pattern of SARS-CoV-2, presenting a new perspective for analyzing the virus through the lens of cancer evolution. In particular, the Omicron variant, a new dominant strain that appeared after the extinction of the Delta variant, was initially undetected for a long period and then reported first in Africa along with numerous accumulated mutations.^[32, 33]^ This phenomenon closely mirrors the punctuated lineage model of cancer evolution. ^[16]^ Based on these aspects, it was possible to metaphorically view the emergence and extinction of numerous SARS-CoV-2 variants, driven by factors such as natural selection, policy interventions, and vaccination, in parallel with the process of potential cancer cells being extinguished by the immune system or relapsing after chemotherapy due to dormant cancer stem cells. A commonality between these two phenomena was that entities not detected through any channels and survived for a long time, accumulating mutations, eventually become dominant (Figure 1). Notably, Omicron has a “long branch”, which can also be called “lost time period”, where mutations accumulated over a long period until being reported worldwide, making it impossible to know the order of occurrence. By integrating phylogenetic information from various regions or continents about this, it was possible to estimate the order of mutation occurrence (Figure 4A’).

Based on the analysis of the global lineage evolution pattern of SARS-CoV-2, it is clear that the virus evolved differently across different continents, with each region exhibiting its unique variant clusters and patterns. The integration of the results from each continent allowed the researchers to identify eight correlated variant clusters, and the worldwide lineage tree was reconstructed based on the occurrence of variants and their relatedness. The reconstructed tree revealed that once all the variants in Cluster 1 occurred, it diverged into two lineages based on the occurrence of specific variants.

The differences in evolutionary patterns between various regions or continents were identified in this article. Since our study only looked at variants according to the WGS of viruses, we do have the limitation on it. It seems reasonable to consider not only viral factors but also host factors to explain them. In this context, the similar distribution of branches in a large pandemic can be seen as an indication of the impact of multiple and non-isolated global communications. Ultimately, however, the difference is likely to be a function of the local, which has much more interaction with each other than the global level of communication. First, vaccination not only prevents the spread of the virus but can also induce a genetic bottleneck. Therefore, it is possible that the patterns between regions where vaccination has been successfully administered and those where it hasn’t slightly show the different patterns of the viral evolution. Particularly, the pace and ratio of vaccination can be highly linked to socioeconomic factors, with economically affluent countries having a higher vaccination rate than those less affluent. On this aspect, several research was reported that high and low vaccine coverage may affect the pattern of the viral evolution based on the conducted simulation or surveillance under such assumptions.^[34, 35, 36]^ Under this background, further investigation is required for a better understanding of the correlation of artificial interventions with evolutionary patterns. Second, it seems to have a significant influence in terms of the viral evolution, such as “the order of isolation.” Strong isolation and quarantine in particular are known to have different initial spread effects that we were unable to capture in our study.^[37]^

We also acknowledge the possibility that the mutation process could have been influenced by transportation by airplanes, vehicles, *etc*.^[38, 39]^ Despite the isolation of each region, we observed that the global pattern of lineage evolution in each region was similar, and the timing of divergence was also similar, indicating a possible interregional influence. Nevertheless, our analysis identified regions with atypical evolutionary trees, such as Senegal, West Africa, and Denmark, which could be potentially due to overall timing or sample bias. Independent pathways and diverging lineages were observed, indicating the complex and dynamic nature of SARS-CoV-2 evolution. Interestingly, the order of variant occurrence within clusters was different across continents, with Africa having a different intra-cluster order than the other three continents. Furthermore, while Asia had a relatively isolated evolutionary lineage, the other continents shared many common evolutionary pathways.

These findings have important implications for understanding the global spread and evolution of SARS-CoV-2. The unique variant clusters and patterns across continents suggest that certain regions may have contributed more to the evolution and spread of the virus than others. This knowledge could be useful in developing targeted interventions to control the spread of the virus in specific regions. Additionally, the identification of independent pathways and diverging lineages highlights the importance of continued genomic surveillance and sequencing efforts to monitor the evolution of SARS-CoV-2 and identify new variants of concern. This knowledge can inform the development of effective vaccines and therapeutics to combat the ongoing pandemic and potential future outbreaks.^[40, 41]^

Our findings suggest that similar to cancer evolution, some evolutionary patterns emerge in the global evolution of SARS-CoV-2 virus. For instance, certain lineages, such as G, GH, GR, GRY, showed neutral or branching evolution, while GK demonstrated typical branching evolution. Furthermore, GRA (omicron), which appears to be typical of punctuated evolution, is noteworthy.^[42, 43]^ Punctuated tumor evolution is characterized by clonal expansion, where early mutations survive clonal sweep by slowly accumulating mutations. In the case of omicrons, many sublineage SARS-CoV-2 viruses accumulated initially undetectable mutations, gradually accumulating variation before suddenly exploding in population to become the dominant species. The presence of relatively many shared mutations indicates that they have survived clonal sweeping for an extended period before reaching their final state. Overall, this interdisciplinary approach to studying viral pathogens through the lens of tumor evolution has the potential to offer new insights into the emergence and spread of viral pathogens, with significant implications for public health and clinical practice.

As we proceeded with our analysis, we observed that certain mutations shared ancestry at different points in time in continents with different hierarchies. For example, we observed mutations like S: S371L, P681H, and orf1ab: R5716C that seemed to form independent pathways and then rejoined in the offspring lineage, and S: A67V, orf1ab: L3674F that diverged and then rejoined in the offspring lineage. Such phenomena are relatively infrequent in human cancer, likely due to the larger genome size of humans. However, in the case of SARS-CoV-2, the relatively small size of its genome may facilitate such occurrences. Nonetheless, the reappearance or repair of specific mutations under certain conditions may indicate a structural tendency of the virus to be mutation-prone, or the existence of conditions that favor their occurrence. Further investigation is required to identify mutation hotspots in these viruses.

In conclusion, the global lineage evolution pattern of SARS-CoV-2 is complex and dynamic, with different regions exhibiting unique clusters and patterns of variants. The integration of results from multiple continents provides a more comprehensive understanding of the evolution of the virus and can inform targeted interventions and genomic surveillance efforts. Overall, these findings have important implications for public health and clinical practice. By studying the evolutionary patterns of SARS-CoV-2, we can gain insight into the emergence and spread of viral pathogens, as well as the development of resistance to antiviral treatments. Additionally, the similarities between tumor evolution and SARS-CoV-2 evolution suggest that new therapeutic approaches developed for cancer may also be applicable to viral diseases.

## Supplementary Material

Supplementary material
