# Supplementary material for: Global lineage evolution pattern of sars-cov-2 in Africa, America, Europe, and Asia: A comparative analysis of variant clusters and their relevance across continents
Source: J Transl Int Med. 2023 Dec 20;11(4):410–22. doi: 10.2478/jtim-2023-0118 (PMC10732492; doi:10.2478/jtim-2023-0118)
Supplement: Supplementary file 1 — Supplementary material [file jtim-2023-0118_sm.pdf]

Supplementary Material

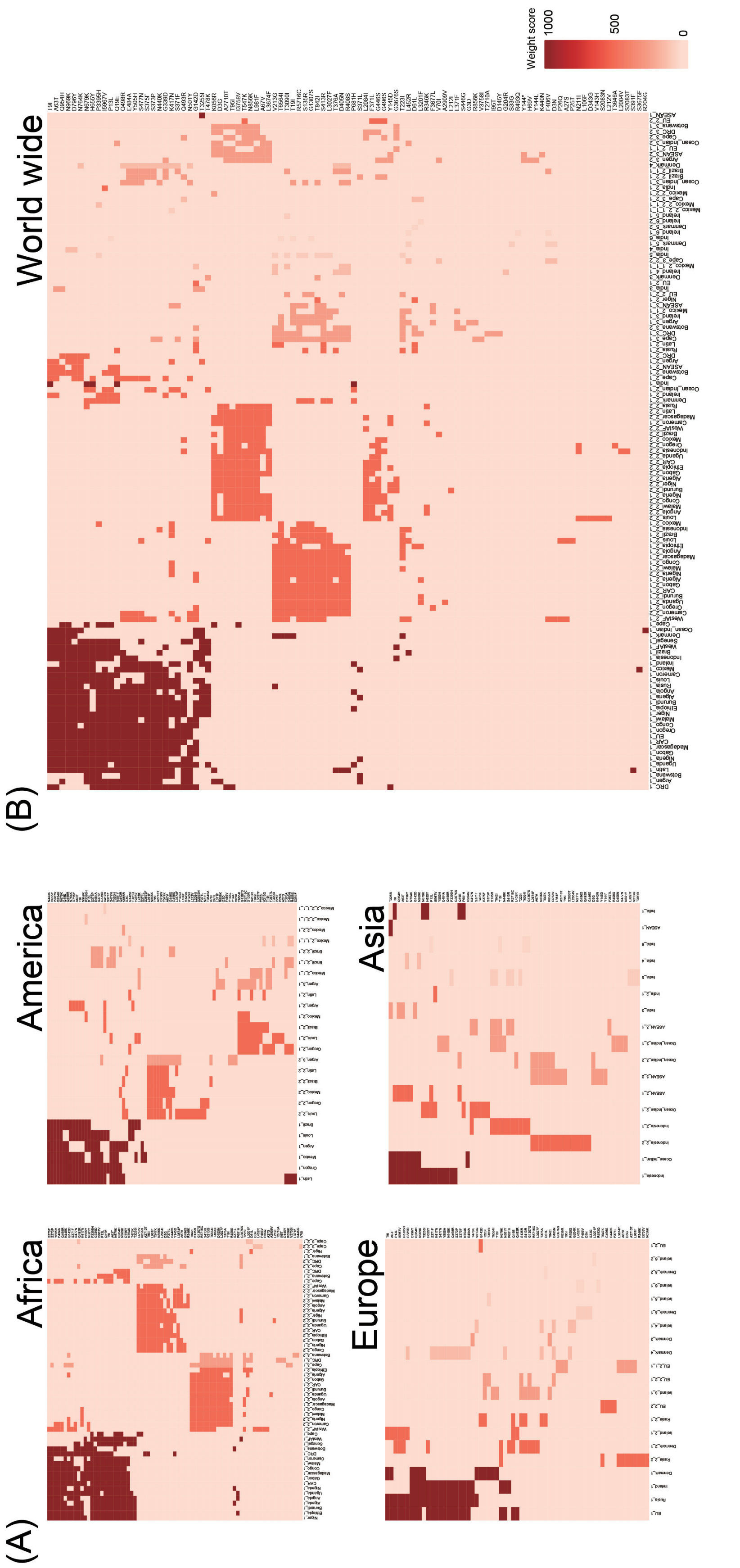

Supplementary Figure 1: Heatmap clustering through weight assignment of phylogenetic tree information. (A, B) Each heatmap was consisted of up to 121 sub-branches and 99 variants information extracted from 32 phylogenetic trees. The number after the country or region name indicates hierarchy of the branches.
